# Supplementary material for: Capturing the temporal evolution of choice across prefrontal cortex
Source: eLife. 2015 Dec 11;4:e11945. doi: 10.7554/eLife.11945 (PMC4718814; doi:10.7554/eLife.11945)
Supplement: Supplementary file 1. — DOI: http://dx.doi.org/10.7554/eLife.11945.023 [file elife-11945-supp1.docx]

**Supplementary Table S1: List of coregressors used when estimating contribution of LFP-derived PC2 to unit activity**

Constant term for effort trials

Constant term for delay trials

Chose left minus chose right

Reward on left option, effort trials, chosen

Reward on left option, delay trials, chosen

Cost on left option, effort trials, chosen

Cost on left option, delay trials, chosen

Reward on right option, effort trials, chosen

Reward on right option, delay trials, chosen

Cost on right option, effort trials, chosen

Cost on right option, delay trials, chosen

Reward on left option, effort trials, unchosen

Reward on left option, delay trials, unchosen

Cost on left option, effort trials, unchosen

Cost on left option, delay trials, unchosen

Reward on right option, effort trials, unchosen

Reward on right option, delay trials, unchosen

Cost on right option, effort trials, unchosen

Cost on right option, delay trials, unchosen
